# 219 Device Stewardship: Opportunities and Experience with Early Efforts

**DOI:** 10.1017/ash.2026.10601

**Published:** 2026-06-23

**Authors:** Abby Griffin, Meghan Maloney, Melissa Morales, Deepa Mavani, Claudia Gutierrez, Kelly Mueller, Manisha Juthani, Lynn Sosa

**Affiliations:** 1 Connecticut Department of Public Health; 2 CT DPH; 3 CT Department of Public Health

## Abstract

**Background:** Hepatitis B is a liver infection caused by the hepatitis B virus (HBV). Outbreaks of HBV infection in healthcare settings have been linked to lapses in infection control, including unsafe injection practices, such as reuse of needles, fingerstick devices, and syringes. In December 2024, an acute case of hepatitis B in a patient without traditional risk factors was reported to the Connecticut Department of Public Health (CTDPH). We investigated a potential healthcare exposure as part of a multidisciplinary investigation. **Methods:** Acute hepatitis B is reportable to CTDPH. Immunization Program staff interviewed the patient with hepatitis B to determine risk factors for infection. Immunization and Healthcare-Associated Infections and Antimicrobial Resistance (HAI-AR) team members assessed potential healthcare exposures via medical chart review. Upon identification of a potential healthcare exposure, HAI-AR staff conducted an on-site Infection Control Assessment and Response (ICAR). **Results:** Patient interview and medical chart review identified potential high-risk procedures at an outpatient clinic that performs aesthetic procedures. Among 15 procedures performed during the six months prior to HBV infection, the highest risk procedures were injection of platelet rich plasma and invasive hormone replacement therapy. ICAR identified the following breaches in infection prevention and control (IPC) practices: poor hand hygiene, failure to label and improper handling of blood products, and improper operation of a sterilizer. Environmental cleaning/disinfection practices were inconsistent, and disinfectants were improperly labeled and stored. HAI-AR staff recommended training on and auditing of hand hygiene, training on proper cleaning/disinfection practices for office staff and environmental services (EVS), and discontinuation of sterilizer use until such time that biologic monitoring could be performed and staff could be properly trained on use. The clinic stopped all operations until IPC oversight was in place, changed all equipment to single-use, and trained staff on disinfection. Providers received bloodborne pathogen training. In response to IPC breaches, the clinic also notified 118 patients who had received high-risk procedures in the prior year to recommend bloodborne pathogen testing. **Conclusion:** Outpatient healthcare environments where aesthetic procedures are performed may have limited regulatory oversight and no requirement for an individual responsible for IPC. In these settings, healthcare providers might perform a large number and variety of invasive procedures, which may require education and training on specialized equipment, such as sterilizers. Improved oversight of these settings with a focus on education and training of outpatient providers on IPC could improve infection prevention and patient safety.

